# Pegylated Recombinant Human Arginase 1 Induces Autophagy and Apoptosis via the ROS-Activated AKT/mTOR Pathway in Bladder Cancer Cells

**DOI:** 10.1155/2021/5510663

**Published:** 2021-03-18

**Authors:** Zhuyun Zhao, Peng Zhang, Wei Li, Dengchuan Wang, Changneng Ke, Yueming Liu, James Chung-Man Ho, Paul Ning-Man Cheng, Shi Xu

**Affiliations:** ^1^Department of Burn and Plastic Surgery, Shenzhen Longhua District Central Hospital, Affiliated Central Hospital of Shenzhen Longhua District, Guangdong Medical University, Shenzhen, Guangdong, China; ^2^Department of Pharmacy, Shenzhen Luohu People's Hospital, Shenzhen, Guangdong, China; ^3^Department of Urology, Shenzhen Longhua District Central Hospital, Affiliated Central Hospital of Shenzhen Longhua District, Guangdong Medical University, Shenzhen, Guangdong, China; ^4^Office of Medical Ethics, Shenzhen Longhua District Central Hospital, Affiliated Central Hospital of Shenzhen Longhua District, Guangdong Medical University, Shenzhen, Guangdong, China; ^5^Division of Respiratory Medicine, Department of Medicine, The University of Hong Kong, Queen Mary Hospital, Pokfulam, Hong Kong SAR, China; ^6^Bio-Cancer Treatment International, 511-513, Bioinformatics Building, Hong Kong Science Park, Tai Po, Hong Kong SAR, China

## Abstract

Bladder cancer is one of the most commonly diagnosed cancers worldwide, especially in males. Current therapeutic interventions, including surgery, radiation therapy, chemotherapy, and immunotherapy, have not been able to improve the clinical outcome of bladder cancer patients with satisfaction. Recombinant human arginase (rhArg, BCT-100) is a novel agent with great anticancer effects on arginine-auxotrophic tumors. However, the effects of BCT-100 on bladder cancer remain unclear. In this study, the *in vitro* anticancer effects of BCT-100 were assessed using four bladder cancer cell lines (J82, SCaBER, T24, and 5637), while the *in vivo* effects were evaluated by establishing T24 nude mice xenograft models. Intracellular arginine level was observed to be sharply decreased followed by the onset of apoptotic events. Furthermore, BCT-100 was found to induce H_2_O_2_ production and mitochondrial membrane depolarization, leading to the release of mitochondrial cytochrome c and Smac to the cytosol. Treatment with BCT was observed to upregulate the expression of LC3B and Becllin-1, but downregulate the expression of p62 in a time-dependent manner. Autophagic flux was also observed upon BCT-100 treatment. Besides, the phosphorylation of the AKT/mTOR pathway was suppressed in a time-dependent fashion in BCT-100-treated T24 cells. While N-acetyl-L-cysteine was shown to alleviate BCT-100-induced apoptosis and autophagy, chloroquine, MK-2206, and rapamycin were found to potentiate BCT-100-triggered apoptosis. Finally, BCT-100 was demonstrated to induce autophagy and apoptosis via the ROS-mediated AKT/mTOR signaling pathway in bladder cancer cells.

## 1. Introduction

Bladder cancer is one of most prevalent diseases worldwide, especially in male. It was estimated that bladder cancer accounts for approximately 3.0% of all new cancer diagnoses and 2.1% of cancer deaths [1]. Urothelial cell carcinoma is the most commonly diagnosed bladder cancer subtype, followed by squamous cell carcinomas [2]. Similar to lung cancer, tobacco use is the leading cause of bladder cancer among other risk factors, which include arsenic exposure, chlorine exposure, and genetics [3–5]. Currently, used standard treatments for bladder cancer patients include surgery, radiation therapy, chemotherapy, and immunotherapy [6, 7]. Although chemotherapy based on the combined use of cisplatin and gemcitabine is currently applied as the first-line treatment strategy for bladder cancer, the emergence of immunotherapy has facilitated the improvement of the overall patient survival rate. However, due to renal insufficiency or cancer cachexia, nearly half of the patients have not been able to benefit from this treatment method [6, 8]. Hence, there is an urgent requirement for the development of novel and safe therapeutic approaches that can improve the clinical outcomes of bladder cancer patients.

Arginine is a semiessential amino acid for human, which exerts multifaceted functions in cellular activities, including cell growth, metabolism, and survival [9]. Arginine is an essential precursor in the synthesis of protein, nitric oxide, polyamines, creatinine, and nucleotides [10]. An in-depth study on the various aspects of tumor metabolism has revealed that metabolic therapy may be a promising treatment option [11]. It has been demonstrated that certain cancer cells are incapable of synthesizing arginine independently, and they are reliant on extracellular arginine for growth [12, 13]. Therefore, arginine deprivation may be a treatment intervention for arginine auxotrophic cancers, including hepatic cell carcinoma, prostate cancer, melanoma, small-cell lung cancer, and leukemia [12–16].

Theoretically, arginine can be metabolized by five enzymatic factors (i.e., nitric oxide synthase, glycine amidinotransferase, arginine decarboxylase, arginine deiminase (ADI), and arginase) to induce arginine depletion. Due to a number of factors, such as drug efficacy, immunogenicity, drug half-time, and potential byproduct generation, only PEGylated ADI and arginase have been employed for the treatment of arginine auxotrophic cancers [17]. An increasing number of studies have shown that two important enzymes in the urea cycle, argininosuccinate synthase (ASS1) and ornithine transcarbamylase (OTC), can greatly affect the efficacy of arginine depletors [13, 18, 19].

In this study, we used BCT-100, which is a classic recombinant human arginase. Consistent with endogenous arginase, BCT-100 catalyzes the conversion of arginine to ornithine and urea, leading to arginine depletion [19]. It has been demonstrated that BCT-100 exhibit significant anticancer effects towards several arginine auxotrophic cancers, and completed clinical trials of BCT-100 on hepatocellular carcinoma (HCC) result in promising outcomes [12, 13, 20]. However, very few studies have investigated the role of BCT-100 on bladder cancer. Here, we explored the underlying mechanisms of arginine deprivation in bladder cancer using BCT-100, which may be used as a potential therapeutic approach.

## 2. Materials and Methods

### 2.1. Cell Lines and Cell Culture

A panel of four bladder cell lines (J82, SCaBER, T24, and 5637), one human bladder cell (SV-HUC-1), and one non-small-cell lung cancer cell line (A549) were purchased from the American Type Culture Collection (Manassas, VA, USA). All cell lines were maintained in RPMI-1640 medium (Gibco, Life Technologies, Carlsbad, California, USA) supplemented with 10% fetal bovine serum (FBS) (Gibco, USA) in a humidified atmosphere containing 5% CO_2_ at 37°C.

### 2.2. Reagents

BCT-100 was kindly provided by Bio-Cancer Treatment International Limited, Hong Kong. N-Acetyl-L-cysteine (NAC, Cat# A7250) and chloroquine (CQ, Cat# C6628) were purchased from Sigma. MK-2206 (Cat# SF2712-5 mg) and rapamycin (Cat# S1842-25 mg) were bought from Beyotime Biotechnology.

### 2.3. Western Blot Analysis

Treated cells were harvested, washed, and resuspended in NP-40 lysis buffer (Beyotime Biotechnology, Jiangsu, China) supplemented with protease inhibitor (1 mM phenylmethylsulphonyl fluoride) for 1 h. Total protein from xenograft samples was extracted using tissue protein extraction reagent kit (Thermo, Cat# 78510). Supernatants were collected after centrifugation (13000 rpm, 4°C, 30 min). For mitochondrial and cytosolic protein extraction, cell mitochondria isolation kit (Beyotime Biotechnology, Cat# C3601) was used. Protein concentration was measured using Bradford Protein Assay Kit (Bio-Rad, Berkeley, CA, USA). Extracted protein (30-60 *μ*g) was separated using 8-15% sodium dodecyl sulfate polyacrylamide gel electrophoresis (SDS-PAGE) and subsequently electroblotted onto polyvinylidene fluoride (PVDF) membranes (Millipore, MA, USA). The membranes were blocked for 1 h at room temperature in 5% nonfat dry milk (dissolved in PBST solution) before being incubated overnight at 4°C with monoclonal or polyclonal primary antibodies (Table 1), followed by incubation with horseradish peroxidase- (HRP-) conjugated secondary antibodies (Cell Signaling Technology, MA, USA) for 1 h at room temperature. Signal detection was conducted using enhanced chemiluminescence (ECL) kit (GE Healthcare). Quantification was performed using GelQuantNET software (Biochem Lab Solutions, CA, USA).

### 2.4. Cell Viability Assay

Cells were seeded in a 96-well plate at a density of approximately 10^4^ cells per well. After drug exposure, cells were incubated with 10 *μ*l of 3-(4,5-dimethylthiazol-2-yl)-2,5-diphenyltetrazolium bromide (MTT; Sigma-Aldrich) at 0.5 mg/ml for 3 h, followed by 100 *μ*l triple lysis solution (10% sodium dodecyl sulfate, 5% isobutanol, and 0.012 mol/l HCl in water) for 2 h. Optical density was measured at 570 nm using FLUOstar OPTIMA microplate reader (BMG Labtech GmbH, Ortenberg, Germany).

### 2.5. Arginine Concentration Detection

The levels of arginine in cells, serum, and tumor were measured using L-arginine ELISA kit (Abcam, Cat# ab241028, MA, USA) according to the manufacturer's instructions. In brief, derivatized samples and standards were incubated with L-arginine antibody overnight at 4°C before being incubated with peroxidase conjugate for 1 h at room temperature and with tetramethylbenzidine substrate for 10 min in the dark. The samples were washed with wash buffer in between each step. Stop solution was added to halt the reaction. Absorbance values at 450 nm (using 620 nm as the reference) were measured using FLUOstar OPTIMA microplate reader (BMG Labtech GmbH, Ortenberg, Germany).

### 2.6. Short Hairpin RNA (shRNA) Transfection

Silencing of ASS1 (shRNA) was conducted using a lentiviral particles kit bought from Santa Cruz (Cat# sc-45810-v). In brief, cells were pretreated with polybrene prior to incubation with lentiviral particles for 24 h. Following the removal of unbound viral particles, cells were further incubated with fresh complete medium for 48 h. Stable ASS1-silenced cell line was selected using puromycin dihydrochloride. The expression of ASS1 protein was evaluated by Western blot after the cells were confirmed to be viable by MTT assay.

### 2.7. Autophagic Flux Analysis

Autophagic flux in T24 cells was detected using mRFP-GFP-LC3 adenovirus (Hanbio, China). Cells were seeded in a 6-well plate at a density of 1 × 10^5^ cells/dish before being incubated with mRFP-GFP-LC3 adenovirus with or without BCT-100 (20 mU/ml) for 24 h. Autophagic flux was observed under an inverted fluorescent microscope (Zeiss, Germany). Yellow and red puncta indicate autophagosomes and autolysosome, respectively.

### 2.8. Flow Cytometry of Annexin V/PI Staining

Apoptosis was determined by flow cytometry using FITC-conjugated Annexin V/PI kit (Beyotime Biotechnology, Jiangsu, China). Briefly, cells were harvested, washed, and resuspended in binding buffer at 5 × 10^6^ ml^−1^. 5 *μ*l of FITC-conjugated Annexin V solution and 10 *μ*l of PE-conjugated PI solution were added to 100 *μ*l of cell suspension. After incubation with Annexin V/PI for 15 min at room temperature, samples were measured using BD FACSAria II analyzer with FL2/FL4 channels (BD, New Jersey, USA). Every sample counted 10000 events for quantification.

### 2.9. Reactive Oxygen Species (ROS) Measurement

The presence of hydrogen peroxide (H_2_O_2_) was tested using 2′,7′-dichlorodihydro-fluorescein diacetate (H2DCFDA; Beyotime Biotechnology, Jiangsu, China). Briefly, treated cells were harvested, washed, and incubated with H2DCFDA (1 *μ*M) in medium without FBS for 30 min at 37°C, followed by washing twice before being analyzed by flow cytometry. The presence of reduced glutathione (GSH) was measured by 5-chloro-methylfluorescein diacetate (CMFDA; Invitrogen, OR, USA). Treated cells were collected, washed, and incubated with CMFDA (5 *μ*M) for 30 min at 37°C in FBS-free medium, following which the medium was changed to complete medium and the cells were further incubated for 30 min prior to flow cytometry analysis.

### 2.10. Detection of Mitochondrial Membrane Depolarization (MMD)

Mitochondrial membrane potential (*ΔΨ*m) was measured using 5,5′,6,6′-tetrachloro-1,1′,3,3′-tetraethyl-benzimidazolylcarbocyanine iodide (JC-1; Beyotime Biotechnology, Jiangsu, China). Briefly, treated cells were incubated in FBS-free medium with JC-1 (5 *μ*g/ml) at 37°C for 15 min in the dark before being washed with PBS. JC-1 fluorescent signal was determined by flow cytometry in FL1/FL2 channels.

### 2.11. Tumor Suppression Effect of BCT-100 Using Xenograft Models

Xenograft models were established by subcutaneous injection of 10^7^ cells into nude mice (4-5 weeks, 10-14 g, female, BALB/cAnN-nu, Laboratory Animal Centre of Shenzhen University, Guangdong, China). When the tumor reached the mean group size of 40–60 mm^3^, mice were randomly divided into three groups. PBS and BCT-100 (20 and 60 mg/kg) were administered intraperitoneally twice a week. Tumor size was calculated according to the formula: (size = length × width × depth/2) [21]. Relative tumor volume (RTV) was calculated by the formula: RTV = *V*_*n*_/*V*_0_, where *V*_*n*_ is tumor size on day *n* and *V*_0_ is tumor size on the first day of treatment. Mice were euthanized using sodium pentobarbital (180 mg/kg) when the tumor volume reached 600 mm^3^, which was regarded as a humane endpoint. The study protocol was approved by the laboratory animal ethical committee of Guangdong Medical University.

### 2.12. Terminal Deoxynucleotidyl Transferase-dUTP Nick End Labeling (TUNEL) Assay

TUNEL assay was conducted using Click-iT Plus TUNEL Assay kit (Beyotime Biotechnology, Jiangsu, China) according to the standard protocol provided by the manufacturer. Cell crawling, fixation, and permeabilization were required before TUNEL assay. Cells were immersed in terminal deoxynucleotidyl transferase (TdT) reaction buffer before being incubated in fresh TdT reaction buffer containing EdUTP, TdT, and TdT enzymes. Samples were incubated with TUNEL reaction cocktails followed by 4′,6-diamidino-2-phenylindole (DAPI, Life Technologies, OR, USA) staining. Images were captured using a Zeiss fluorescence microscope.

### 2.13. Immunofluorescence

Fixed and permeabilized cells or tumor sections were blocked with 3% bovine serum albumin (BSA) for 1 h at room temperature, followed by incubation with ki67 antibody overnight at 4°C. After washing with PBST for 30 min, Alexa Fluor anti-rabbit (Life Technologies, OR, USA) antibody was applied for 1 h in the dark. Slides were mounted with ProLong Gold antifade reagent and DAPI. Images were obtained using a Zeiss fluorescence microscope.

### 2.14. Statistical Analysis

All data were obtained from at least three independent experiments and are shown as the mean ± standard deviation (S.D.). Statistical analysis was performed using Student's two-tailed *t*-test by Prism 5 (GraphPad Software, La Jolla, Southern California, USA).

## 3. Results

### 3.1. Sensitivity of Bladder Cancer Cells to BCT-100 Correlated with the Expression of ASS1 and OTC

**Figure 1:**
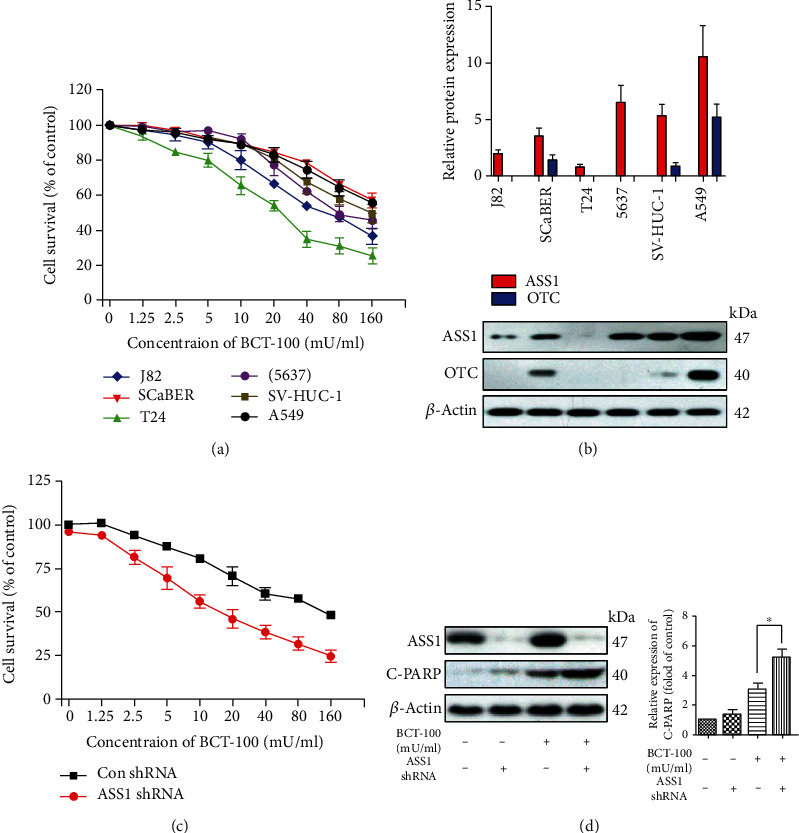
Sensitivity of bladder cancer cells to BCT-100 correlated with the expression of ASS1 and OTC. (a) Cell growth inhibition evaluated by MTT assay in bladder cancer cell lines (J82, SCaBER, T24, and 5637), human bladder cell line (SV-HUC-1), and A549 cells treated with BCT-100 for 72 h. (b) Basal expression levels of ASS1 and OTC in bladder cancer cells evaluated by Western blot. (c) Cell survival rate of ASS1-silenced 5637 cells (72 h) evaluated by MTT assay. (d) Expression of C-PARP and ASS1 in ASS1-silenced 5637 cells treated with BCT-100 for 72 h. *β*-Actin was used as a loading control.

Firstly, the cell viability of four bladder tumor cell lines, including three transitional cell carcinomas (J82, T24, and 5637) and one squamous cell carcinoma (SCaBER), one human bladder cell line (SV-HUC-1), and one lung cancer cell line (A549, positive control) treated with increasing concentrations of BCT-100 for 3 days, was evaluated by MTT assay. As shown in Figure 1(a), T24 and SCaBER cells had the highest (IC_50_ = 20 mU/ml) and the lowest (IC_50_ = 200 mU/ml) level of sensitivity to BCT-100 treatment, respectively. Next, the basal protein expression of ASS1 and OTC, two of the most important enzymes for arginine biosynthesis in the urea cycle, was evaluated by Western blot. ASS1 expression was relatively high in A549 and 5637 cells, whereas OTC was highly expressed in SCaBER and A549 cells (Figure 1(b)).

In order to explore the role of ASS1 upon BCT-100 exposure, ASS1 was knocked down in 5637 cells using lentiviral shRNA vector. Our results showed that knockdown of ASS1 increased the sensitivity of 5637 cells to BCT-100 (Figure 1(c)), and that the expression of cleaved poly ADP-ribose polymerase (C-PARP) was upregulated in ASS1-silenced cells after BCT-100 exposure for 3 days (Figure 1(d)).

Since T24 cells were shown to be relatively sensitive to BCT-100 treatment, this cell line was selected as the model cell line in the following experiments to study the underlying mechanism of anticancer effect of BCT-100 in bladder cancer.

### 3.2. BCT-100 Induced Cytotoxicity and Lowered the Level of Arginine in Bladder Cancer Cells

**Figure 2:**
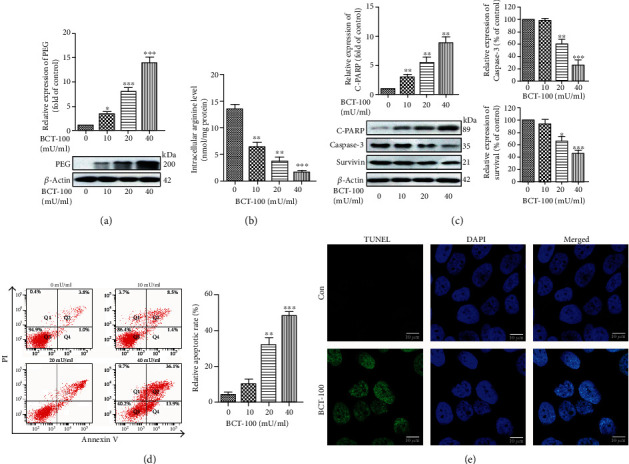
BCT-100 induced cytotoxicity and lowered the level of arginine in bladder cancer cells. (a, c) Intracellular PEG, Caspase 3, Survivin, and C-PARP evaluated by Western blot in T24 cells exposed to BTC-100 (20 mU/ml) for 72 h. (b) Intracellular arginine levels following BCT-100 treatment for 72 h. (d) Apoptotic rates determined by Annexin V and PI staining analyzed by flow cytometry in cells treated with BCT-100 (20 mU/ml) for 72 h. (e) TUNEL assay indicating the presence of apoptotic cells exposed to BCT-100 (20 mU/ml) for 72 h. Data are represented as the mean ± S.D. of three independent experiments.^∗^*p* < 0.05, ^∗∗^*p* < 0.01, and ^∗∗∗^*p* < 0.001, as evaluated by Student's *t*-test.

BCT-100, a derivative of recombinant human arginase conjugated to polyethylene glycol (PEG), is used to indicate the accumulation and localization of arginase *in vitro* and *in vivo*. While PEG was detected in T24 cells upon BCT-100 exposure in a dose-dependent manner (Figure 2(a)), intracellular arginine concentration was significantly decreased in a dose-dependent manner (Figure 2(b)). Meanwhile, cleaved PARP (C-PARP) was upregulated, and Caspase 3 and Survivin were downregulated, which was an indication of cytotoxicity induced by BCT-100 treatment (Figure 2(c)). Annexin V/PI staining (Figure 2(d)) and TUNEL assay (Figure 2(e)) showed an increase in the number of apoptotic cells. The apoptotic rate of T24 cells treated with BCT-100 at 20 mU/ml and 40 mU/ml was increased sharply to 32.0 ± 6.3% and 48.0 ± 4.1%, respectively, compared to that of untreated cells (4.0 ± 2.4%).

### 3.3. Effect of BCT-100 on ROS Production, Mitochondrial Membrane Depolarization, and Mitochondria-Dependent Apoptosis

**Figure 3:**
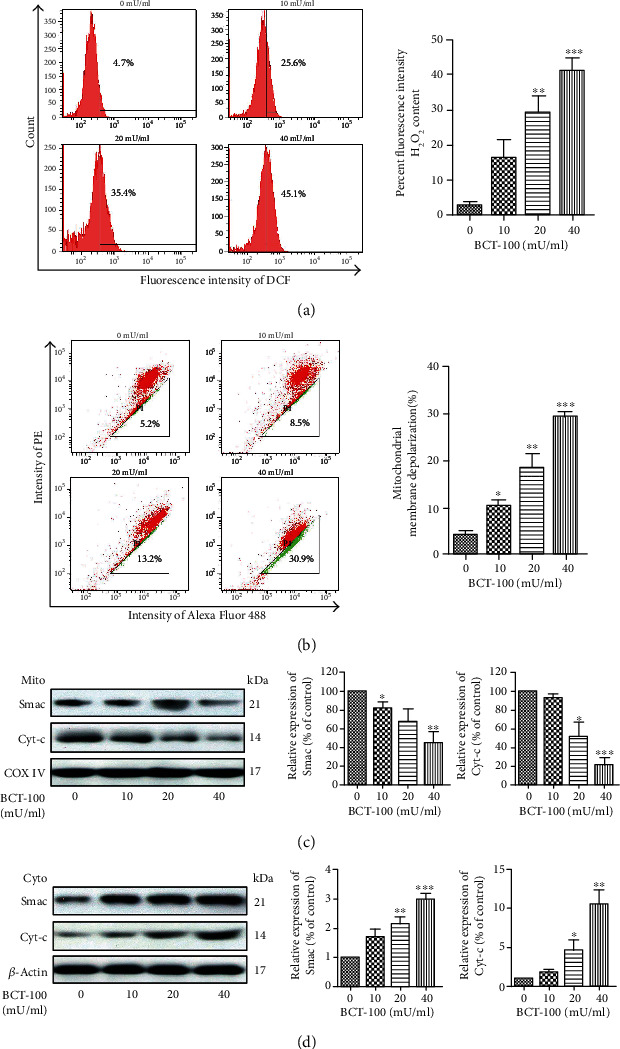
Apoptosis induced by BCT-100 was mitochondrial-dependent and accompanied by ROS production. (a) Elevation of H_2_O_2_ after BCT-100 treatment (20 mU/ml) for 24 h analyzed by flow cytometry. (b) Mitochondrial membrane depolarization shown by JC-1 staining in T24 cells upon BCT-100 (20 mU/ml) exposure for 24 h. (c, d) Subcellular localization of Cyt-c and Smac assessed by Western blot in cells treated with BCT-100 (20 mU/ml) for 72 h. COX IV and *β*-actin were used as loading controls in mitochondrial and cytosolic fraction, respectively. Data are represented as the mean ± S.D. of three independent experiments.^∗^*p* < 0.05, ^∗∗^*p* < 0.01, and ^∗∗∗^*p* < 0.001, as evaluated by Student's *t*-test.

Intracellular hydrogen peroxide (H_2_O_2_) was detected using fluorescence probe DCFH-DA. As shown in Figure 3(a), the production of H_2_O_2_ was significantly induced upon BCT-100 exposure for 24 h. The relative percentage of DCF fluorescence intensity was boosted from 2.5 ± 1.9% (control) in untreated cells to 16.1 ± 8.3% (10 mU/ml), 29.1 ± 8.1% (20 mU/ml), and 41.0 ± 6.3% (40 mU/ml) in BCT-100-treated cells. The level of reduced glutathione (GSH), as indicated by CMFDA staining, was observed to be decreased in a dose-dependent manner upon BCT-100 treatment for 24 h (Figure S1). Since mitochondria are essential for the production of ROS, mitochondrial membrane potential was measured using JC-1 staining. As shown in Figure 3(b), mitochondrial membrane depolarization occurred in T24 cells upon BCT-100 exposure for 3 days. The percentage of JC-1-positive cells in the control, 10 mU/ml, 20 mU/ml, and 40 mU/ml group was 4.1 ± 1.6%, 10.3 ± 2.1%, 18.5 ± 5.0%, and 29.3 ± 1.8%, respectively. Next, the subcellular localization of Smac and cytochrome c (Cyt-c), both playing a crucial role in mitochondrial-dependent apoptosis, was investigated. As shown in Figures 3(c) and 3(d), while the levels of mitochondrial Smac and Cyt-c were decreased in cells treated with BCT-100 in a dose-dependent fashion, the levels of cytosolic Smac and Cyt-c were upregulated.

### 3.4. BCT-100 Induced Autophagy and Activated the AKT/mTOR Pathway

**Figure 4:**
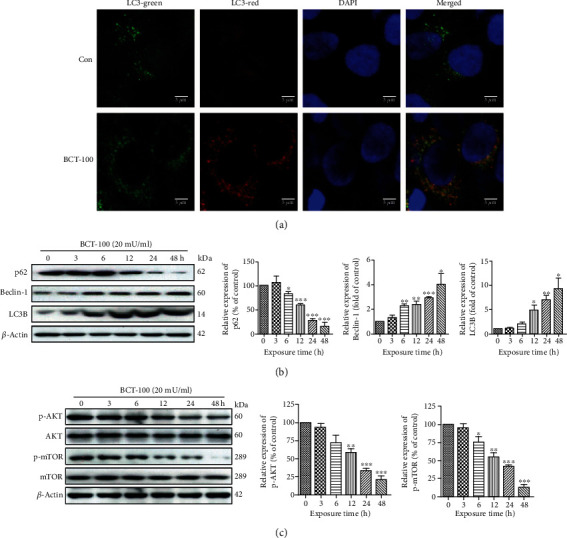
Autophagy induction and AKT/mTOR pathway activation after BCT-100 exposure. (a) T24 cells were transfected with the mRFP-GFP-LC3 adenovirus for 24 h and treated with BCT-100 (20 mU/ml) for another 24 h. Representative pictures of fluorescent LC3 puncta were captured by an inverted fluorescent microscope. (b, c) BCT-100-treated T24 cells were harvested at different time points. LC3B, p62, Beclin-1, p-AKT, AKT, p-mTOR, mTOR, and *β*-actin were tested by Western blot. All data are presented as the mean ± S.D. of three independent experiments.^∗^*p* < 0.05, ^∗∗^*p* < 0.01, and ^∗∗∗^*p* < 0.001, as evaluated by Student's *t*-test.

To evaluate the extent of autophagy induced by BCT-100, T24 cells were transfected with mRFP-GFP-LC3 adenovirus. As displayed in Figure 4(a), the number of red and yellow (merged) puncta was remarkably increased in BCT-100-treated cells. Additionally, upon BCT-100 stimulation, the expression levels of autophagy-related biomarkers, Beclin-1 and LC3B, were upregulated in a time-dependent manner, whereas the expression level of p62 was downregulated (Figure 4(b)). Since the AKT-mTOR pathway exerts important roles in cell proliferation and autophagy, the phosphorylation levels of AKT and mTOR in BCT-100-treated cells were evaluated. As shown in Figure 4(c), the phosphorylation of both AKT (Ser473) and mTOR (Ser2448) was inhibited in a time-dependent fashion.

### 3.5. BCT-100-Induced ROS Initiated Cellular Apoptosis and Autophagy in Bladder Cancer Cells

**Figure 5:**
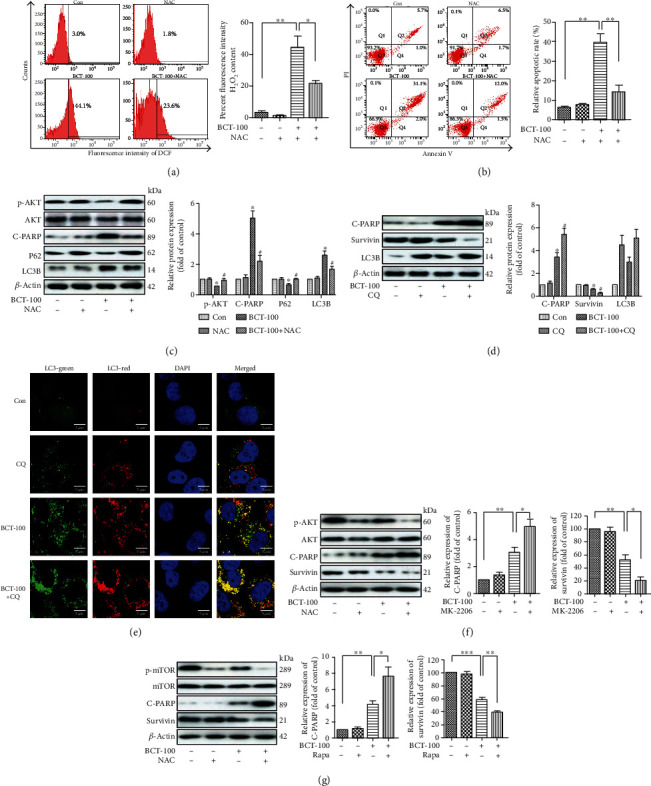
BCT-100-induced ROS initiated cellular apoptosis and autophagy in bladder cancer cells. (a) ROS levels determined by flow cytometry in T24 cells pretreated with NAC (5 mM, 1 h) before incubation with BCT-100 (20 mU/ml) for 24 h and H2DCFDA (1 *μ*M). (b) Cell apoptotic rate determined by flow cytometry of Annexin V/PI staining in T24 cells pretreated with NAC (5 mM, 1 h) before incubation with BCT-100 (20 mU/ml) for 72 h. (c) Expression of AKT, p-AKT, C-PARP, p62, LC3B, and *β*-actin evaluated by Western blot. (d) Expression of C-PARP, Survivin, LC3B, and *β*-actin evaluated by Western blot in T24 cells treated with BCT-100 (20 mU/ml) and CQ (10 *μ*M) for 3 days. (e) Autophagic flux assessed by confocal microscopy in mRFP-GFP-LC3-transfected T24 cells treated with BCT-100 (20 mU/ml) and CQ (10 *μ*M) for 3 days. (f, g) Expression levels of related proteins tested by Western blot in cells treated with MK-2206 (2 *μ*M) and rapamycin (100 nM) with or without BCT-100 for 3 days. *β*-Actin was used as a loading control. All data are shown as the mean ± S.D. of three independent assays. ^∗^*p* < 0.05 versus control and ^#^*p* < 0.05 versus BCT-100, as evaluated by Student's *t*-test.

Our results above indicated that BCT-100 exposure in cells induced the production of ROS, which is a key regulator of cellular apoptosis and autophagy. To investigate the role of ROS in BCT-100-mediated apoptosis and autophagy, we employed NAC, an effective ROS scavenger in our study. As shown in Figure 5(a), cellular ROS level was significantly decreased in cells treated with NAC and BCT-100 compared to that in cells treated with BCT-100 alone (21.5 ± 3.3% vs. 44.2 ± 12.4%). Meanwhile, the apoptotic rate of cells treated with NAC and BCT-100, compared to those treated with BCT-100 alone, was 14.1 ± 5.9% and 39.6 ± 7.4%, respectively (Figure 5(b)). Moreover, NAC inhibited the expression of C-PARP as well as LC3B, but promoted the expression of p62 in combination arm (Figure 5(c)). Next, to clarify the role of autophagy in cells treated with BCT-100, CQ, an autophagy inhibitor was recruited. As shown in Figure 5(d), cellular apoptosis was significantly induced upon treatment with CQ and BCT-100, since the expression levels of C-PARP and Survivin were observed to be upregulated and downregulated, respectively, in the combination group. Consistently, cells exposed to CQ and BCT-100 showed increased accumulation of autolysosome (red puncta) and autophagosome (yellow puncta; Figure 5(e)). Furthermore, specific AKT and mTOR inhibitors (MK-2206 and rapamycin) were found to potentiate BCT-100-induced cytotoxicity, as indicated by the increased C-PARP expression coupled with decreased Survivin expression in cells exposed to CQ and BCT-100 (Figures 5(f) and 5(g)).

### 3.6. BCT-100 Suppressed Tumor Growth in T24 Xenograft Models

**Figure 6:**
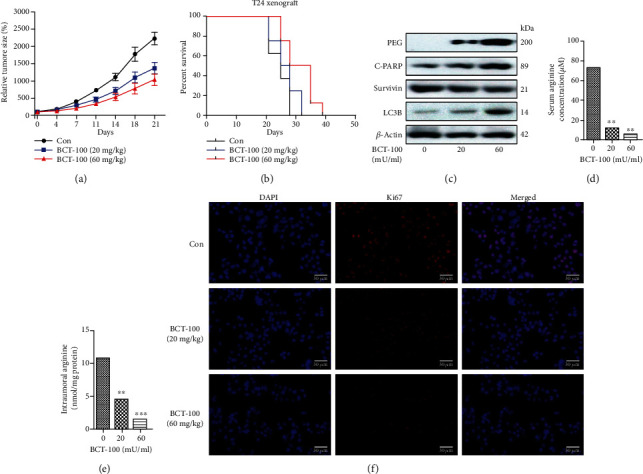
BCT-100 suppressed tumor growth *in vivo*. (a) Relative tumor volume and (b) median survival of mice in the mock-treated and BCT-100-treated groups (20 mg/kg and 60 mg/kg) recorded in different time points (*n* = 7 − 8 per group). (c) Expression of PEG, LC3B, C-PARP, and Survivin assessed by Western blot. Serum (d) and intratumoral (e) arginine levels in mice were detected by L-arginine kit. (f) Immunofluorescence staining of Ki67 in mock-treated and BCT-100-treated mice. Bar, 30 *μ*m. All data are presented as the mean ± S.D. of three independent experiments.^∗^*p* < 0.05, ^∗∗^*p* < 0.01, and ^∗∗∗^*p* < 0.001, as evaluated by Student's *t*-test.

The tumor growth rate was observed to be significantly inhibited in mice receiving BCT-100 (60 mg/kg) treatment compared to that in mock-treated mice (Figure 6(a)). In addition, the median survival of mice was prolonged following BCT-100 treatment from 25.0 days (mock-treated control group) to 26.5 days (20 mg/kg treatment group) and to 31.5 days (60 mg/kg treatment group; Figure 6(b)). Next, the accumulation of PEG, LC3B, and apoptotic biomarkers in BCT-100-treated mice was investigated. In line with our *in vitro* results earlier, the levels of accumulated PEG, LC3B, and C-PARP were observed to be increased sharply, whereas the level of Survivin was found to be decreased (Figure 6(c)). Meanwhile, the concentrations of serum arginine in mice from the control group, 20 mg/kg treatment group, and 60 mg/kg treatment group were found to be 72.7 ± 14.4 *μ*M, 11.8 ± 3.5 *μ*M, and 5.6 ± 2.6 *μ*M, respectively (Figure 6(d)). Similarly, the levels of intratumoral arginine in mice were observed to be declined dramatically from 10.8 ± 1.1 *μ*M (control group) to 4.7 ± 0.5 *μ*M (20 mg/kg treatment group) and to 1.6 ± 0.3 *μ*M (60 mg/kg treatment group; Figure 6(e)). Finally, the fluorescence intensity of Ki67, a biomarker associated with cell proliferation, was found to be decreased in a dose-dependent manner in mice treated with BCT-100 (Figure 6(f)).

## 4. Discussion

In this study, PEGylated recombinant human arginase 1 (BCT-100) was shown to sharply deplete the levels of intracellular and intratumoral arginine and display an antiproliferative effect on tumor growth in bladder cancer cells. The underlying mechanisms were demonstrated to mainly involve apoptosis and autophagy, which are related to the oxidative stress-activated AKT/mTOR cell signaling pathway. In bladder cancer xenograft models, a high dose of BCT-100 (60 mg/kg) was found to remarkably inhibit tumor growth and prolong median survival.

Due to the dysregulation in cellular metabolism, cancer cells require more nutrients to support their proliferation. Therefore, amino acid deprivation has been considered as a promising approach in cancer therapy. Arginine, asparagine, glutamine, methionine, and serine are important amino acids for the tumor growth of certain auxotrophic cancers [17]. One of the most successful examples is L-asparaginase-induced asparagine depletion used in the treatment of acute leukemia. Similarly, arginine depletion therapy has recently been recognized as another encouraging therapeutic strategy in cancer treatment.

As a targeted metabolic anticancer therapy, the efficiency of arginine deprivation is related to the expression of ASS1 and OTC, the two key enzymes in arginine biosynthesis [22]. Previous studies have reported that ASS1- and/or OTC-deficient cell lines are relatively vulnerable to arginine depletors [13, 19]. For instance, it has been illustrated that ASS1 silencing can enhance the sensitivity of small-cell lung cancer (SCLC) cells to ADI treatment [18, 23]. Notably, it has been demonstrated that, while knockdown of ASS1 in H69 cells (SCLC) with or without BCT-100 treatment exhibits no significant differences, blocking of OTC sensitizes H841 cells (SCLC) to BCT-100 exposure [13]. Consistently, in our study, we observed that knockdown of ASS1 increases the sensitivity of 5637 cells to BCT-100 treatment (Figures 1(c) and 1(d)). BCT-100 was also observed to display selective anticancer effects on tumor cells, since nontumorigenic cell line (HaCaT cell), compared with T24 cell line, was found to be relatively resistant to BCT-100 exposure (Figure S2). Nevertheless, knockdown of OTC was observed to be unable to increase the sensitivity of SCaBER cells to BCT-100 (data not shown). This may be due to the weak basal expression of OTC in SCaBER cells, and there may be other noncanonical pathways that regenerate arginine. However, further studies are needed to support our view.

The underlying mechanisms of arginine depletors might vary in different cancer cell types. ADI, the most well-studied arginine depletory agent, has been shown to induce mitochondrial dysfunction, ROS production, and autophagy in prostate cancer [24]. The Warburg effect was involved after ADI treatment in leiomyosarcoma and melanoma [25]. Akin to ADI in cancer therapy, rhArg has also been demonstrated to exhibit tumor inhibition effects through autophagy induction, oxidative stress, and cell cycle arrest in breast cancer, hepatocellular carcinoma, and acute myeloid leukemia [19, 26, 27]. Therefore, it is necessary to discuss the mechanism of BCT-100 in bladder cancer cells for better clinical outcomes.

Reactive oxygen species (ROS) homeostasis is critical to maintaining the biological functions of cells. Dysregulation of ROS production and metabolism has been shown to cause oxidative stress, which is responsible for several pathological processes, such as autophagy, apoptosis, inflammation, DNA damage, aging, and neurological diseases [28]. The common forms of ROS are the superoxide anion (O_2_^−^) and hydrogen peroxide (H_2_O_2_) and their derived forms, hydroxyl radical (HO·) and hydroperoxyl radical (HOO·). Numerous chemotherapeutic drugs have been found to exert antitumor effects via ROS-dependent cytotoxicity in cancer cells *in vitro* and *in vivo* [29–31]. In our study, ROS production was boosted and glutathione (GSH) was correspondingly decreased in a dose-dependent fashion after BCT-100 stimulation. Besides, the ROS scavenger NAC could eliminate ROS level thus restore the cell viability. Mitochondrion is an important organelle in various biological activities, including energy generation, signal transduction, cell differentiation, and apoptosis [28]. Since mitochondrion is one of the major sources of ROS, we postulated that BCT-100 can affect mitochondrial function and induce mitochondrial-dependent apoptosis. Consistent with our previous findings, JC-1 staining assay revealed the occurrence of mitochondrial membrane depolarization (MMD) upon BCT-100 treatment. Concurrently, upon mitochondrial stress, Cyt-c along with Smac is released from mitochondria to cytosol, indicating the initiation of mitochondrial-mediated apoptosis. Taken together, mitochondrial-dependent cytotoxicity due to excessive ROS production is regulated by BCT-100 in bladder cancer cells.

Autophagy is an important cellular event in which cytoplasmic materials are directly degraded by lysosomes [32]. Although cancer cells benefit from autophagy under stressful conditions, such as nutrient deprivation, hypoxia, and oxidative stress, excessive autophagy activities may lead to cell death [33]. Due to the two-faced role of autophagy in cancer therapy as a savior or an executioner, it is necessary to determine the function of autophagy in bladder cancer cells after BCT-100 treatment. In this study, our findings revealed that BCT-100 led to upregulation of Beclin-1 and LC3B, downregulation of p62, and the occurrence of autophagic flux, suggesting autophagy was activated. The autophagy inhibitor CQ was shown to potentiate BCT-100-triggered cytotoxicity, implying that autophagy plays a protective role in BCT-100-treated T24 cells. Consistent with our findings, the prosurvival role of autophagy induced by rhArg has been also demonstrated in laryngeal squamous cell carcinoma [34]. However, autophagy activated by HuArgI (Co)-PEG5000 has been shown to cause autophagic cell death in pancreatic cancer cells [35]. The AKT-mTOR signaling pathway plays an important role in the regulation of apoptosis and autophagy. Activation of AKT-mTOR signaling pathway has been shown to stimulate cell growth. In the present study, BCT-100 was shown to inhibit the phosphorylation of AKT and mTOR, without affecting the total protein level, suggesting that the AKT-mTOR signaling pathway is mediated by BCT-100. In addition, NAC was observed to significantly recover the phosphorylation of AKT and subsequently restore cell viability. Besides, both MK-2206 and rapamycin were shown to enhance the cytotoxicity induced by BCT-100, implying that the AKT-mTOR signaling pathway plays a crucial role in the underlying mechanism.

It is essential to study the *in vivo* anticancer effects of BCT-100 using xenograft models, which offer a systemic effect with the microenvironment of tumors being present under arginine deprivation. BCT-100 was observed to suppress tumor growth and prolong the survival time with arginine depletion by inducing tumoral apoptosis. The BCT-100 dosage (20 mg/kg and 60 mg/kg) used in this study was relatively low in comparison with that in other malignant tumor models [26, 36], due to the tumor susceptibility to BCT-100 treatment. Furthermore, the body weight of nude mice in medication groups did not markedly decrease contrasted with the control group (data not shown), indicating the side effect of BCT-100 might be acceptable. The most common adverse reactions of cisplatin- and gemcitabine-treated cancer bladder patients include vomiting, nephrotoxicity, and myelosuppression [37, 38]. It may be an option for BCT-100 to be combined with chemotherapeutics to diminish the side effects. Since patients receiving BCT-100 in preclinical trials show good tolerance, BCT-100 may be a good alternative therapeutic agent in the clinical management of bladder cancer.

Many clinical trials of rhArg have been initiated in cancer patients, including patients with leukemia, melanoma, HCC, and prostate cancer. The early-phase clinical trial of rhArg has been completed in patients with HCC. It has been shown that the conditions of HCC cancer patients with adequate arginine depletion intervention exhibit significant improvement compared to with those with less arginase treatment (median progression-free survival, 6.4 vs. 1.7 months, 95% CI: 1.67-1.73, *p* = 0.01) [39]. A study on ADI clinical trials has demonstrated that ADI can prolong progression-free survival in patients with ASS1-deficient mesothelioma (3.2 vs. 2.0 months), and that the overall patient survival in the ADI and control group is 15.7 and 12.3 months, respectively [40]. The side effects of arginine deprivation therapy have been observed to be relatively mild, which include liver dysfunction, neutropenia, fatigue, and nausea [20, 40]. However, formal phase III clinical trial of ADI on advanced HCC patients has not shown an overall survival benefit in the second-line treatment [41]. Further clinical trials of arginine deprivation in cancer patients are still underway.

Nevertheless, there are several limitations in our study. NAC, CQ, and rapamycin were not employed to confirm the corresponding functions *in vivo*. It would be more reasonable to use PEG as the normal control in our study, since BCT-100 is a PEG-modified rhArg, although PEG is known to be inert to cells [42]. Besides, it is necessary to find out noncanonical pathways to regenerate arginine, which may provide convincing evidence to support our hypothesis.

## 5. Conclusions

In conclusion, we demonstrated that recombinant human arginase (BCT-100) display good anticancer effects against bladder cancer via apoptosis and autophagy regulated by the ROS-activated AKT/mTOR signaling pathway. Our study provides a scientific ground that may facilitate the future clinical development of recombinant human arginase for treating bladder cancer.

## Figures and Tables

**Table 1:** List of primary antibodies used in this study.

Name	Species	Manufacturer	Cat#	Molecular weight (kDa)	Dilution factor
ASS1	Rabbit	Santa Cruz Biotechnology	SC-99178	47	1 : 500
C-PARP	Rabbit	Santa Cruz Biotechnology	SC-7150	89	1 : 1000
PEG	Rabbit	RevMAb Biosciences	31-1008-00	200	1 : 1000
OTC	Rabbit	Santa Cruz Biotechnology	SC-102051	40	1 : 200
m-TOR	Rabbit	Cell Signaling Technology	2972	289	1 : 1000
p-mTOR	Rabbit	Cell Signaling Technology	5526	289	1 : 1000
Survivin	Rabbit	Cell Signaling Technology	2808	16	1 : 1000
Smac	Mouse	Cell Signaling Technology	2954	21	1 : 1000
Cyt-c	Rabbit	Cell Signaling Technology	4280	14	1 : 1000
COX IV	Mouse	Cell Signaling Technology	11967	17	1 : 1000
AKT	Rabbit	Cell Signaling Technology	9272	60	1 : 1000
p-AKT	Rabbit	Cell Signaling Technology	4060	60	1 : 1000
p62	Mouse	Cell Signaling Technology	88588	62	1 : 1000
LC3B	Rabbit	Cell Signaling Technology	3868	14	1 : 1000
Beclin-1	Rabbit	Cell Signaling Technology	3495	60	1 : 1000
Caspase 3	Rabbit	Cell Signaling Technology	9662	35	1 : 1000
*β*-Actin	Mouse	Sigma-Aldrich	A5441	42	1 : 5000

## Data Availability

The data used to support the findings of this study are included within the article.

## Abbreviations

BCT-100: Bio-Cancer Treatment 100
PEG: Polyethylene glycol
C-PARP: Cleaved poly (ADP-ribose) polymerase
ASS1: Argininosuccinate synthetase
OTC: Ornithine transcarbamylase
ADI: Arginine deiminase
ROS: Reactive oxygen species
Smac: Second mitochondria-derived activator of caspases.

## Supplementary Materials

Supplementary 1 Supplementary Figure S1: GSH content after BCT-100 treatment.

Supplementary 2 Supplementary Figure S2: the effect of BCT-100 on HaCaT and T24 cell.
