# Supplementary figures and images for: Pegylated Recombinant Human Arginase 1 Induces Autophagy and Apoptosis via the ROS-Activated AKT/mTOR Pathway in Bladder Cancer Cells
Source: Oxid Med Cell Longev. 2021 Mar 18;2021:5510663. doi: 10.1155/2021/5510663 (PMC7996046; doi:10.1155/2021/5510663)

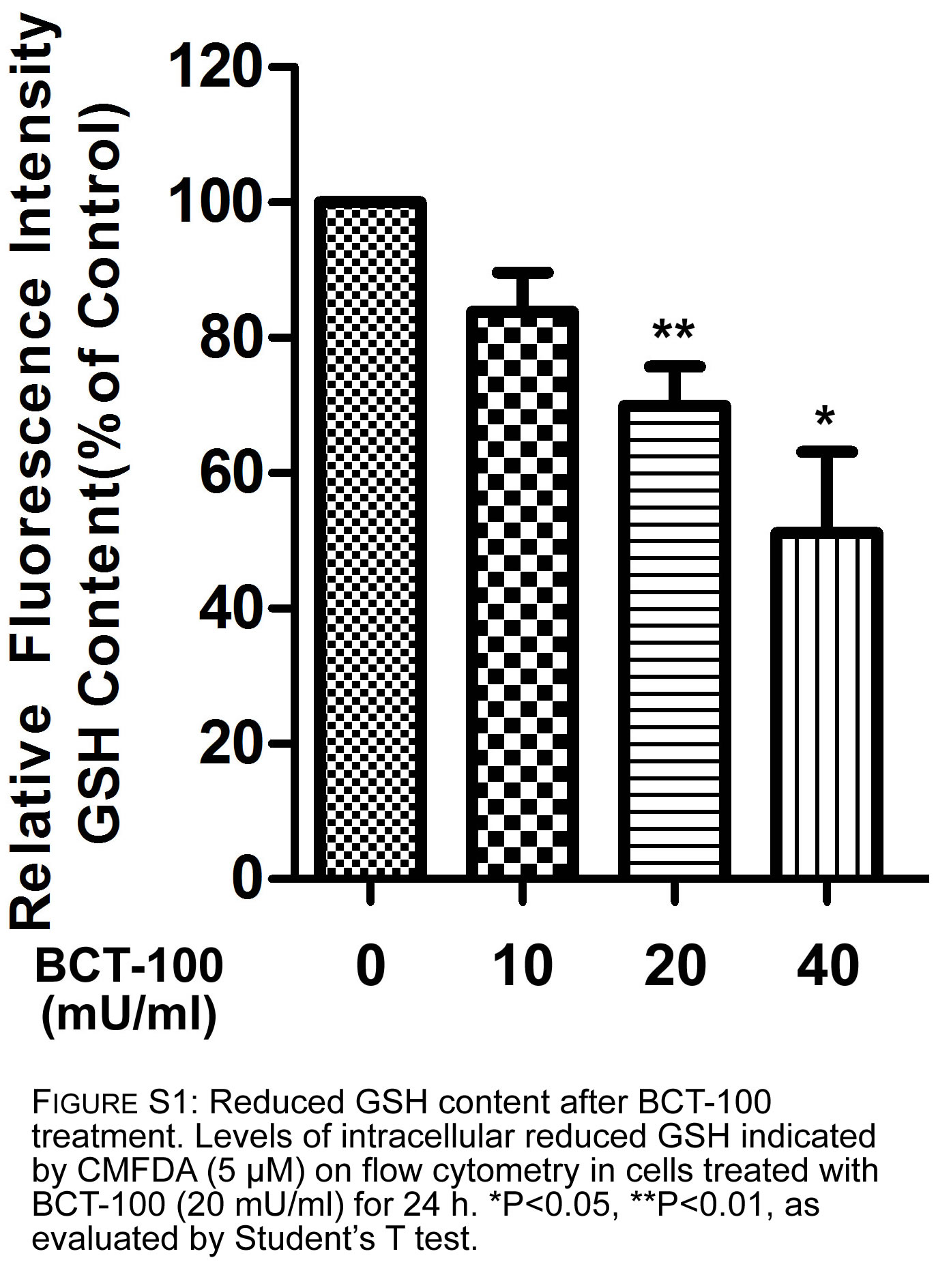

Supplement: Supplementary 1 — Supplementary Figure S1: GSH content after BCT-100 treatment. [file 5510663.f1.jpg]

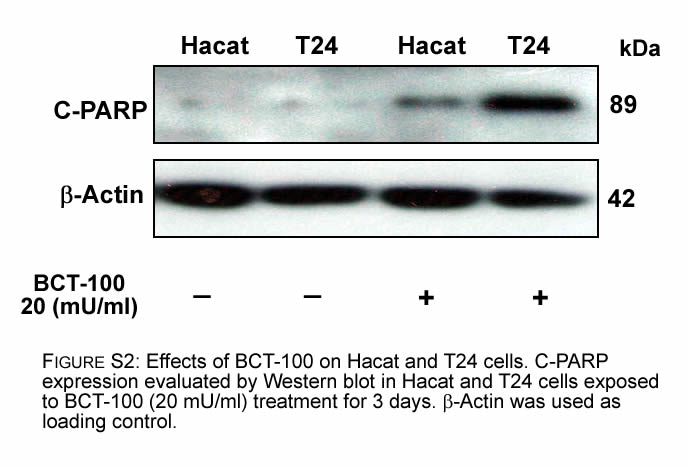

Supplement: Supplementary 2 — Supplementary Figure S2: the effect of BCT-100 on HaCaT and T24 cell. [file 5510663.f2.jpg]
